# A Novel *In Vitro* Method for Detecting Undifferentiated Human Pluripotent Stem Cells as Impurities in Cell Therapy Products Using a Highly Efficient Culture System

**DOI:** 10.1371/journal.pone.0110496

**Published:** 2014-10-27

**Authors:** Keiko Tano, Satoshi Yasuda, Takuya Kuroda, Hirohisa Saito, Akihiro Umezawa, Yoji Sato

**Affiliations:** 1 Department of Reproductive Biology, National Research Institute for Child Health and Development, Tokyo, Japan; 2 Division of Cellular & Gene Therapy Products, National Institute of Health Sciences, Tokyo, Japan; 3 National Research Institute for Child Health and Development, Tokyo, Japan; 4 Department of Quality Assurance Science for Pharmaceuticals, Graduate School of Pharmaceutical Sciences, Nagoya City University, Aichi, Japan; 5 Department of Cellular & Gene Therapy Products, Graduate School of Pharmaceutical Sciences, Osaka University, Osaka, Japan; 6 Department of Translational Pharmaceutical Sciences, Graduated School of Pharmaceutical Sciences, Kyushu University, Fukuoka, Japan; Wake Forest Institute for Regenerative Medicine, United States of America

## Abstract

Innovative applications of cell therapy products (CTPs) derived from human pluripotent stem cells (hPSCs) in regenerative medicine are currently being developed. The presence of residual undifferentiated hPSCs in CTPs is a quality concern associated with tumorigencity. However, no simple *in vitro* method for direct detection of undifferentiated hPSCs that contaminate CTPs has been developed. Here, we show a novel approach for direct and sensitive detection of a trace amount of undifferentiated human induced pluripotent stem cells (hiPSCs) using a highly efficient amplification method in combination with laminin-521 and Essential 8 medium. Essential 8 medium better facilitated the growth of hiPSCs dissociated into single cells on laminin-521 than in mTeSR1 medium. hiPSCs cultured on laminin-521 in Essential 8 medium were maintained in an undifferentiated state and they maintained the ability to differentiate into various cell types. Essential 8 medium allowed robust hiPSC proliferation plated on laminin-521 at low cell density, whereas mTeSR1 did not enhance the cell growth. The highly efficient culture system using laminin-521 and Essential 8 medium detected hiPSCs spiked into primary human mesenchymal stem cells (hMSCs) or human neurons at the ratio of 0.001%–0.01% as formed colonies. Moreover, this assay method was demonstrated to detect residual undifferentiated hiPSCs in cell preparations during the process of hMSC differentiation from hiPSCs. These results indicate that our highly efficient amplification system using a combination of laminin-521 and Essential 8 medium is able to detect a trace amount of undifferentiated hPSCs contained as impurities in CTPs and would contribute to quality assessment of hPSC-derived CTPs during the manufacturing process.

## Introduction

Cell therapy products (CTPs) are expected to offer promising treatments for serious and life-threating diseases for which no adequate therapy is currently available. An increasing number of CTPs derived from human pluripotent stem cells (hPSCs), i.e. induced pluripotent stem cells (hiPSCs) and embryonic stem cells (hESCs), are being developed for regenerative medicine/cell therapy because of their infinite self-renewal capacity and their ability to differentiate into various types of cells. Quality assessment of CTPs is critical to ensure their safety and efficacy for clinical application [1]. CTPs derived from hPSCs possibly include the cells of interest and also other cells such as undifferentiated cells, precursor cells and other differentiated cells. The presence of residual undifferentiated cells in CTPs derived from hPSCs is one of the most serious concerns for tumorigenicity because the undifferentiated hPSCs have a capacity to form teratoma in animals [1]–[4]. Hentze et al. previously reported that hundreds of undifferentiated hESCs were enough to produce a teratoma in immunodeficient SCID mice [5]. We cannot exclude the possibility that a trace amount of residual undifferentiated hPSCs in CTPs cause ectopic tissue formation, tumor development and/or malignant transformation after transplantation. Therefore, establishment of a detection method for residual undifferentiated cells is necessary for the safety and quality assessment of CTPs derived from hPSCs.

An *in vivo* teratoma formation assay is the only method to directly assess tumorigenicity of undifferentiated cells, but this assay is costly and time-consuming [2], [3]. Several *in vitro* methods, such as flow cytometry and quantitative real-time PCR (qRT-PCR) analysis, can also detect residual undifferentiated hPSCs in CTPs [2], [3]. Our previous report has shown that flow cytometry using anti-TRA-1-60 antibody and qRT-PCR using a specific probe and primers for *LIN28* mRNA can detect as low as 0.1% and 0.002% undifferentiated hiPSCs spiked into retinal pigment epithelial (RPE) cells, respectively [3]. However, both of these methods have the disadvantage of detecting undifferentiated cell marker expression but not functionally undifferentiated cells *per se*. The soft agar colony formation assay is commonly used to detect tumorigenic cells with a property of anchorage-independent growth. However, this assay is not appropriate for the detection of hPSCs because they undergo apoptosis associated with dissociation into single cells [3], [6]. At present, there is no simple method to directly detect a trace amount of hPSCs *in vitro*.

Recently, some cell culture matrices have been reported to sustain self-renewal of dissociated hPSCs without apoptosis [7], [8]. We focused on a culture system enabling hPSC cell growth without apoptosis and developed a direct *in vitro* method for detecting a trace amount of undifferentiated hPSCs in CTPs. Laminin-521, a laminin isoform that is normally expressed in hESCs, is known to stimulate robust hPSC proliferation in an undifferentiated state in combination with mTeSR1 medium [7]. In the present study, we present a novel approach to detect undifferentiated hiPSCs contaminating CTPs through efficient amplification using a laminin-521-based cell culture system with Essential 8 medium [9] instead of mTeSR1 medium.

## Materials and Methods

### Cell culture

The hiPSC lines, 201B7, 253G1 and 409B2, were provided by the RIKEN BRC through the Project for Realization of Regenerative Medicine and the National Bio-Resource Project of the MEXT, Japan [17]–[19]. hiPSCs were first cultured on mitomycin C-treated SNL cells (a mouse fibroblast STO cell line expressing a neomycin-resistance gene cassette and LIF) in primate ES cell medium (ReproCell, Kanagawa, Japan) supplemented with 4 ng/ml human basic fibroblast growth factor (bFGF; R&D Systems, Inc., Minneapolis, USA). hiPSC colonies were passaged as small clumps once every 5–6 days using CTK solution (ReproCell) and STEMPRO EZPassage (Invitrogen, Carlsbad, CA, USA). hiPSCs were then passaged onto Matrigel-coated dishes with mTeSR1 (Stem Cell Technologies, Vancouver, CAN) for at least 2 passages before plating on laminin-521 or directly subcultured onto laminin-521-coated dishes. Subculture on laminin-521-coated dishes was performed as follows: near-confluent cells were treated with 0.5 mM EDTA/D-PBS for 6–7 minutes at 37°C. Cells were pipetted to achieve single-cell suspension and centrifuged at 30× *g* for 4 minutes. After centrifugation, the cell pellet was suspended in Essential 8 medium (Life Technologies, USA) and seeded at 2−3×10^4^ cells/cm^2^ on laminin-521-coated dishes. Cells were grown in Essential 8 medium at 37°C in a 5% CO_2_ atmosphere and passaged once in 3–4 days. Primary human mesenchymal stem cells (hMSCs) were purchased from Lonza and cultured in MSCGM medium (Lonza, Walkersville, MO, USA). hMSCs at passage 7 were used in this study. Primary human neurons were purchased from ScienCell Research Laboratories (Carlsbad, CA, USA).

### Cell proliferation assay

hiPSCs were dissociated into single cells and seeded on matrix-coated plates at a density of 3×10^4^ cells/cm^2^ or at the indicated density as described below. Tissue culture plates (BD Falcon, NJ, USA) were coated with laminin-521 (BioLamina, Sundbyberg, Sweden) dissolved in D-PBS at 4 µg/cm^2^ at 37°C for 2 h. Control plates were coated with Matrigel (BD Biosciences, MA, USA) at 16 µg/cm^2^. Viable cells were quantified every 24 h using CyQUANT Cell Proliferation Assay Kit (Life Technologies) according to the manufacturer's instructions.

### Quantitative RT-PCR

Total RNA was treated with DNase I and isolated using RNeasy Mini Kit (Qiagen Hilden, Germany) according to the manufacturer's instructions. Quantitative RT-PCR was performed using the QuantiTect Probe one-step RT-PCR Kit (Qiagen) on StepOnePlus Real Time PCR system (Life Technologies). Gene expression levels were normalized to *GAPDH* expression levels, which were quantified using TaqMan human GAPDH control reagents (Life Technologies). Primers and probes were obtained from Sigma-Aldrich. The sequences of primers and probes are listed in Table S1.

### Teratoma assay

Teratoma formation experiments were performed by injecting 253G1 cells (1×10^6^ cells/testis) that were cultured with laminin-521 and Essential 8 medium (passage 36), into the testes of severe combined immunodeficiency (SCID) mice at the age of 8 weeks under pentobarbital anesthesia. The mice were sacrificed with an overdose of pentobarbital 10 weeks after the transplant, and the isolated teratoma was fixed in 10% formalin. The paraffin-embedded section was stained with hematoxylin and eosin (HE). Animal experiments were performed at UNITECH Co., Ltd. (Chiba, Japan) in accordance with the animal ethical committee's approval (Permit Number: KIS-130712i-20 at UNITECH Co., Ltd. and 444 at NIHS).

### Differentiation assay

Differentiation of hiPSCs into three germ layers was performed as follows: 253G1 cells were plated on laminin-521 at a density of 3×10^4^ cells/cm^2^ in Essential 8 medium and expanded until they were nearly confluent. A) Ectoderm lineage differentiation: Neural hiPSCs differentiation was performed according to the previously reported protocol with some modifications [10]. Briefly, culture medium was changed from Essential 8 to DMEM/F12 medium containing 20% knockout serum replacement (KSR, Life Technologies), 10 µM SB431542 (Sigma-Aldrich) and 500 ng/ml Noggin (R&D systems). After 4 days of differentiation, SB431542 was withdrawn and increasing amounts of N2 medium (25%, 50%, or 75%) was added to the KSR medium every 2 days. From day 10 of differentiation, the medium was changed to N2B27 medium without bFGF containing 500 ng/ml Noggin, and cells were cultured for 15 days. B) Mesoderm lineage differentiation: hiPSCs were cultured for 15 days in DMEM/F12 containing 10% FBS, 2 mM _L_-glutamine 1% nonessential amino acids (Life Technologies) and 0.1 mM β-mercaptoethanol [8]. C) Endoderm lineage differentiation: hepatic differentiation of hiPSCs was performed according to the previously reported protocol with some modifications [11]. On the first day of differentiation, the medium was replaced with RPMI1640 (Sigma-Aldrich) containing B27 supplement (Life Technologies), 100 ng/ml activin A (R&D systems), 50 ng/ml Wnt3a (R&D systems) and 1 mM sodium butyrate (NaB) (Sigma-Aldrich). On the following 2 days, NaB was omitted from the medium. After 3 days of differentiation, the medium was replaced with knockout-DMEM containing 20% KSR, 1 mM _L_-glutamine, 1% nonessential amino acids, and 1% DMSO for 5 days.

Differentiation of 253G1 cells into MSCs was performed according to the previously reported protocol with some modifications [14]. On the first day of differentiation, 253G1 cells subcultured on laminin-521 in Essential 8 medium were dissociated into single cells and suspended in EB formation medium (AggreWell Medium, Stem Cell Technologies) with 10 µM Y-27632 (Wako, Japan), a ROCK inhibitor for generation of embryoid bodies (EBs). Cells (1×10^6^) were then added to a well of the AggreWell Plate and incubated for 24 h at 37°C in a 5% CO_2_ atmosphere. After 24 h, EBs were plated on 35-mm dishes (BD) in Stemline II (Sigma-Aldrich) supplemented with 50 ng/ml BMP4 (R&D systems) and 50 ng/ml VEGF (R&D systems) for 2 days. Medium was changed to Stemline II containing BMP4 (50 ng/ml), VEGF (50 ng/ml) and bFGF (22.5 ng/ml), and the EBs were cultured for 2 days. EBs were dissociated into single cells and replated in Methocult H4536 (Stem Cell Technologies) containing Growth Enhancement Media Supplement (EX-CYTE, Millipore), 50 ng/ml VEGF, 50 ng/ml Flt3-ligand (R&D systems), 50 ng/ml thrombopoietin (R&D systems) and 30 ng/ml bFGF for hemangioblast formation. After 8 days, cultures were harvested and plated as defined passage 0 in MSC growth medium (αMEM+20% FBS) on Matrigel.

### Immunofluorescence staining

Immunofluorescence staining was performed as follows: cells were fixed with 4% paraformaldehyde in PBS for 15 minutes. After washing three times with PBS, cells were permeabilized with 0.1% Triton X-100 in PBS for 10 minutes, and then blocked with Blocking One (nacalai tesque, Kyoto, Japan) at 4°C over night. Cells were incubated with primary antibody against α-fetoprotein (AFP, 1∶400; Dako) for 30 minutes at room temperature, with antibody against smooth muscle actin (SMA, 1∶400; Sigma-Aldrich), βIII tubulin (0.5 µg/ml; abcam) or TRA-1-60 (1∶200; Millipore) for 1 hour at room temperature, or with antibody against CD105 (1∶200; abcam) at 4°C over night. After washing with PBS three times, cells were incubated with secondary antibody conjugated with Alexa Fluor 488 (Invitrogen) for 30 minutes at RT. VECTASHIELD mounting medium with DAPI (VECTOR) was used for nuclear staining. The samples were examined using an Olympus IX71 microscope equipped with cellSens Standard software (Olympus).

### Embryoid body formation

Embryoid bodies were generated from hiPSCs using AggreWell 800 plates (Stem Cell Technologies) according to the manufacturer's instructions, with some modifications. hiPSCs dissociated with 0.5 mM EDTA were collected and suspended in EB formation medium (AggreWell Medium, Stem Cell Technologies) supplemented with 10 µM Y-27632 (Wako). The cells were added to each well (5×10^5^ cells/well) in the AggreWell plate and incubated for 24 h at 37°C in a 5% CO_2_ atmosphere. EBs were harvested from AggreWell plate and cultured in 35-mm dishes (BD) with primate ES cell culture medium (ReproCell) without bFGF. The medium was changed every 3 days. After 10 days of incubation, total RNA was isolated from EBs. The expression levels of each differentiation marker were determined using quantitative RT-PCR, as described above.

### Statistics

Statistical analysis was performed using SigmaPlot 12.5 Software (Systat Software Inc., CA). The data were analyzed using two-way ANOVA or two-way repeated-measures ANOVA followed by a Bonferroni t-test as a post hoc test. A probability below 0.05 was considered significant.

## Results

### Essential 8 medium promotes hiPSCs cell growth on laminin-521

**Figure 1:**
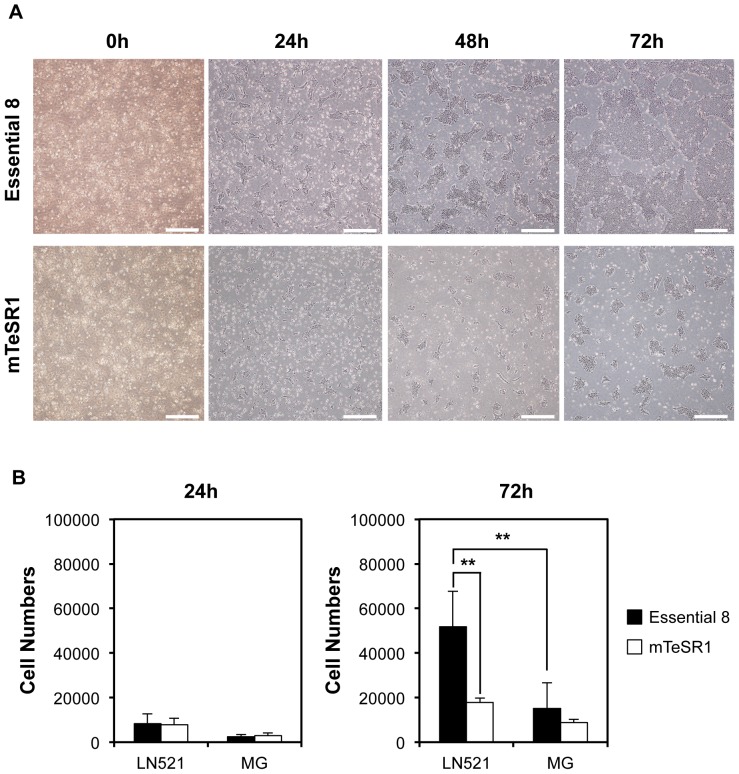
Robust proliferation of 253G1 cells cultured on laminin-521 in Essential 8 medium. (A) Morphology of the 253G1 cells expanded on laminin-521 in Essential 8 or mTeSR1 medium after dissociation into single cells. Scale bars, 500 µm. (B) Quantification of the number of dissociated 253G1 cells expanded on laminin-521 or Matrigel in Essential 8 or mTeSR1 medium. Data are presented as the mean ± standard deviation (SD) of three independent experiments (**P<0.01, two-way ANOVA followed by Bonferroni t-test as post-hoc test). LN521, laminin-521; MG, Matrigel.

hPSCs are known to easily undergo apoptosis induced by dissociation [6]. To achieve an efficient hPSC cell growth, we determined the optimal culture conditions that allow robust proliferation of hiPSCs dissociated into single cells. Here, we focused on laminin-521 as a cell culture matrix, which permits survival of dissociated hPSCs without a Rho-associated protein kinase (ROCK) inhibitor [7]. mTeSR1 medium is conventionally used to culture dissociated hiPSCs on dishes coated with laminin-521 [7], and other hPSC media besides mTeSR1 have not been fully characterized with laminin-521. To examine effects of medium on hPSC cell growth on laminin-521, we compared the hiPSC growth rate using conventional mTeSR1 medium with Essential 8 medium, with optimized components [9]. After subculture on Matrigel, 253G1 hiPSCs were dissociated into single cells and seeded on laminin-521-coated plates at a density of 3×10^4^ cells/cm^2^. No difference was observed between mTeSR1 and Essential 8 medium in the cell number of hiPSCs cultured on laminin-521 at 24 h after plating (Figure 1A and 1B). However, the cells cultured on laminin-521 in Essential 8 medium showed rapid expansion compared to those in the mTeSR1 medium, and they reached nearly confluent at 72 h after plating (Figure 1A). Cell growth quantification revealed that the number of cells in Essential 8 medium was 3-fold higher than those in mTeSR1 medium at 72 h after plating (Figure 1B). Similar results were also obtained using another hiPSC line, 201B7 (Figure S1A). These results suggest that Essential 8 medium promotes hiPSC proliferation more rapidly than mTeSR1 medium when grown on laminin-521. When dissociated hiPSCs were cultured on Matrigel, Essential 8 medium did not significantly promote cell proliferation compared with mTeSR1 medium (Figure 1B and Figure S1A). We found that Essential 8 medium enhanced the hiPSC growth rate even on LM511-E8, which is a fragment of laminin 511 [8], but this effect was weaker than that on laminin-521 (Figure S1B). Taken together, these results suggest that a combination of laminin-521 and Essential 8 medium is a potent cell culture system for efficient amplification of dissociated hiPSCs *in vitro* compared to other culture systems. Therefore, we decided to develop a novel method for detecting undifferentiated hPSCs using a combination of laminin-521 and Essential 8 medium.

### Culture system using laminin-521 and Essential 8 medium maintains the undifferentiated state and pluripotency of hiPSCs

**Figure 2:**
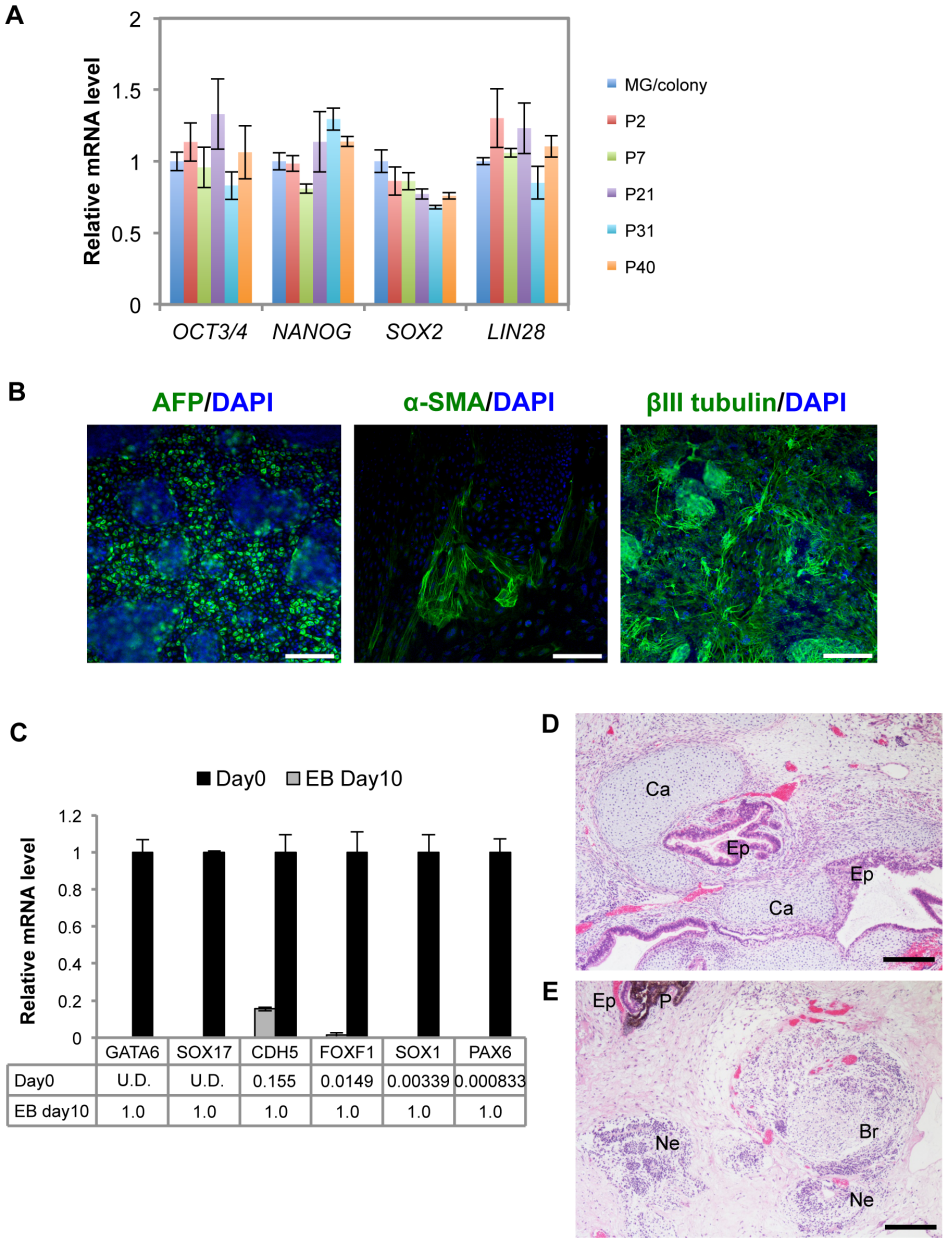
Characterization of 253G1 cells subcultured on laminin-521 in Essential 8 medium. (A) Expression levels of undifferentiated cell markers (*OCT3/4, NANOG, SOX2 and LIN28*) in 253G1 cells subcultured on laminin-521 in Essential 8 were determined using qRT-PCR. Relative mRNA expression levels are presented as ratios to the level of that in control cells on Matrigel. Results are the mean ± SD (n = 3). (B) *In vitro* differentiation analysis of 253G1 cells subcultured on laminin-521 in Essential 8 medium. Immunostaining of the markers for three germ layers are shown: endoderm (alpha-fetoprotein (AFP)), mesoderm (α-smooth muscle actin (SMA)) and ectoderm (βIII tubulin). Scale bars, 200 µm. (C) Expression levels of differentiated cell markers in embryoid bodies (EBs) derived from 253G1 cells: endoderm (*GATA6, SOX17*), mesoderm (*CDH5, FOXF1*), ectoderm (*SOX1, PAX6*). Relative mRNA expression levels are presented as ratios to the level of that in control cells (EBs at Day 10). Results are the mean ± SD (n = 3). (D-E) Teratomas derived from 253G1 cells cultured on laminin-521 in Essential 8 medium are shown. Hematoxylin and eosin staining showed the features of three germ layers: Ep, epithelium-like tissue (endoderm); Ca, cartilage (mesoderm); Ne, neural rosette-like tissue (ectoderm); P, pigmented neuroectodermal resembling meranocyte (ectoderm); Br, brain-like tissue (ectoderm). Scale bars, 200 µm.

We next examined whether a culture system using laminin-521 and Essential 8 medium maintains undifferentiated states through serial passages. 253G1 cells were dissociated into single cells and sequentially subcultured on laminin-521 in Essential 8 medium. 253G1 cells exhibited vigorous proliferation for more than 30 passages under these conditions (data not shown). Quantitative RT-PCR analysis revealed that the expression levels of undifferentiated hPSC markers (*OCT3/4*, *NANOG*, *SOX2* and *LIN28*) in 253G1 cells cultured with laminin-521 and Essential 8 medium were similar to those cultured with Matrigel and mTeSR1 medium (Figure 2A). Moreover, the serial passages with laminin-521 and Essential 8 medium did not have any effect on expression of the undifferentiated markers. We also examined the effects of subculture on laminin-521 in Essential 8 medium using other hiPSC lines, 201B7 and 409B2, and showed that these cells expressed undifferentiated markers through serial passages (Figure S2A-B). We next tested the pluripotency of hiPSCs cultured on laminin-521 in Essential 8 medium using lineage-specific differentiation protocols. Immunofluorescence analysis using antibodies specific for markers of three germ layers clearly demonstrated that 253G1 cells subcultured with laminin-521 and Essential 8 medium could selectively differentiate into endoderm, mesoderm and ectoderm expressing AFP, α-SMA and βIII tubulin, respectively (Figure 2B). To further examine pluripotency of hiPSCs subcultured with laminin-521 and Essential 8 medium, we facilitated spontaneous differentiation of cells grown as aggregates (embryoid bodies). Embryoid bodies derived from 253G1 cells increased gene expression of differentiated markers for all three germ layer lineages (Figure 2C), consistent with the observation obtained using 201B7 and 409B2 cells (Figure S2C-D). We also confirmed that 253G1 cells cultured with laminin-521 and Essential 8 medium were engrafted in testes of SCID mice and formed teratomas that involved all three germ layers (Figure 2D). These results strongly suggest that a culture system using laminin-521 and Essential 8 medium supports the undifferentiated state and pluripotency of hiPSCs through serial passages.

### Essential 8 medium enables hiPSCs to proliferate rapidly from low cell density on laminin-521

**Figure 3:**
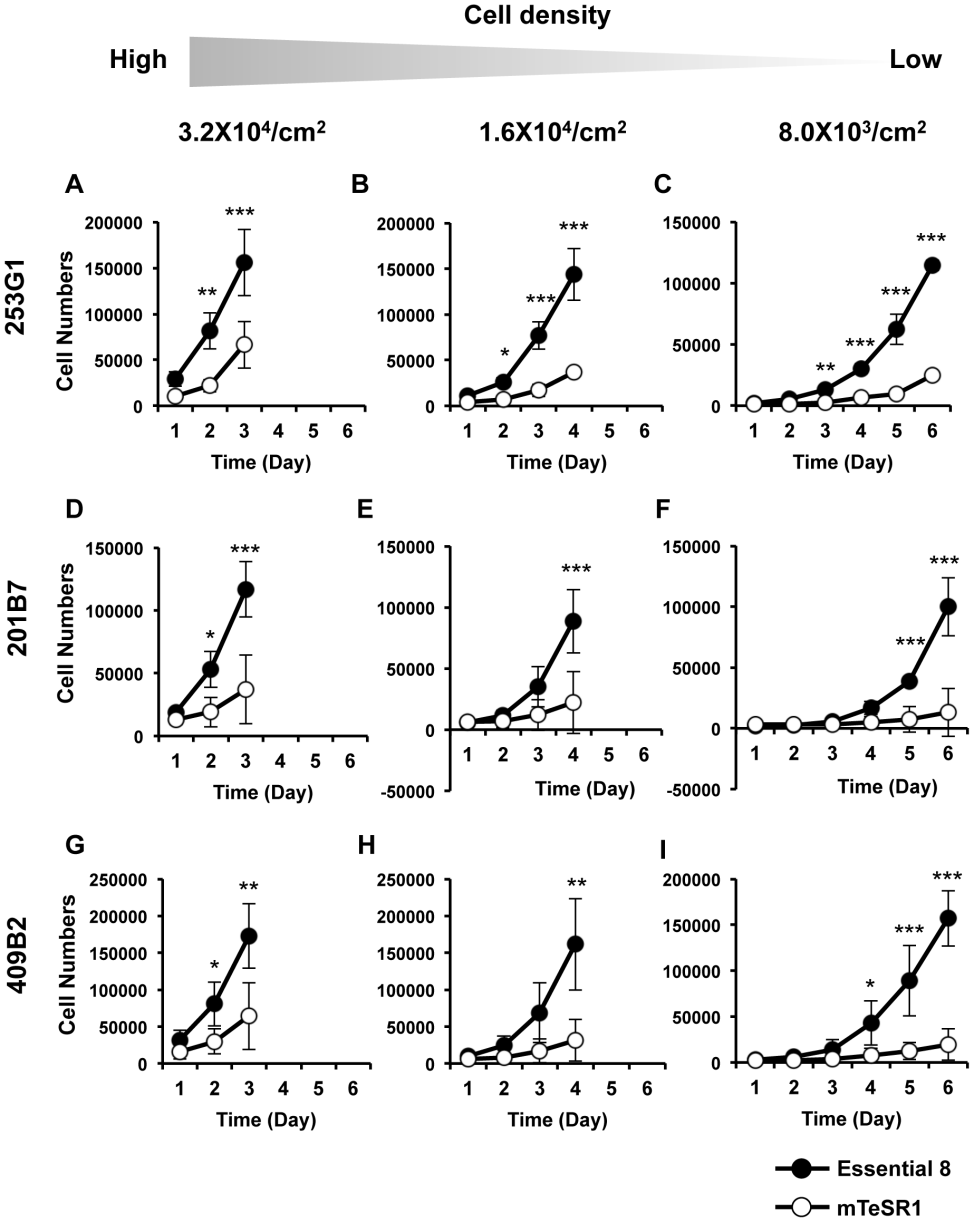
Rapid cell proliferation of hiPSCs plated at low cell density on laminin-521 in Essential 8 medium. (A-I) Quantification of the number of 253G1, 201B7 and 409B2 cells expanded on laminin-521 in Essential 8 or mTeSR1 medium. Cell numbers were counted every 24 h after plating at 3.2×10^4^ cells/cm^2^ (A, D, G), 1.6×10^4^ cells/cm^2^ (B, E, H) and 8.0×10^3^ cells/cm^2^ (C, F, I), respectively. Data are presented as the mean ± standard deviation (SD) of three independent experiments (*P<0.05, **P<0.01, *** *P*<0.001, two-way repeated-measures ANOVA followed by a Bonferroni post-hoc test).

Higher sensitivity is expected to ensure the accuracy and reliability of the detection of trace amounts of undifferentiated cells. To achieve higher sensitivity, culture system using laminin-521 and Essential 8 should have a capacity to rapidly expand hPSCs even at a low cell density. Therefore, we next tested whether Essential 8 medium promotes expansion of the hiPSCs on laminin-521 plated at a low cell density. 253G1 cells were seeded into laminin-521-coated plates at a density of 3.2×10^4^ cells/cm^2^, 1.6×10^4^ cells/cm^2^ and 8.0×10^3^ cells/cm^2^, and grown until nearly confluent. Cells grown in mTeSR1 reduced proliferative capacity as seeding density became lower. Conversely, cells cultured in Essential 8 medium showed robust propagation over a prolonged period of time even when they were seeded at low cell density (Figure 3A-C). Similarly, 201B7 and 409B2 cells seeded at lower density in Essential 8 medium also showed robust proliferation compared to the cells in mTeSR1 (Figure 3D-I). Essential 8 medium also promoted cell growth when hiPSCs were plated at lower density of 800 cells/cm^2^ (Figure S3). These results indicate that Essential 8 medium promotes expansion of hiPSCs plated on laminin-521 at a low cell density. Thus, a culture system using laminin-521 and Essential 8 medium is considered to be well suited for direct detection of trace amounts of undifferentiated cells.

### Culture system using laminin-521 and Essential 8 medium is useful for direct detection of undifferentiated hiPSCs contained in somatic cells

**Figure 4:**
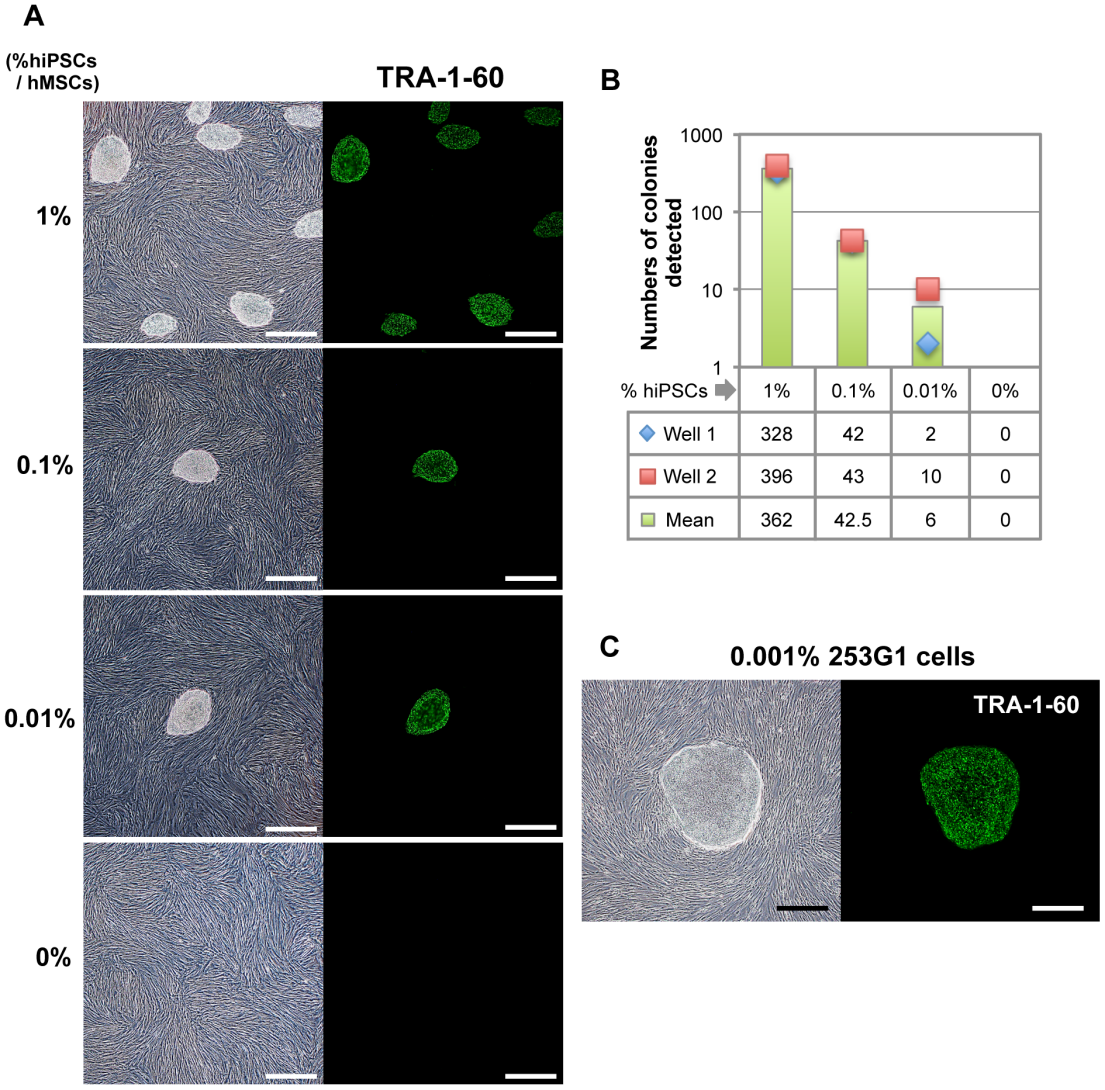
Detection of hiPSCs spiked into hMSCs on the culture system using laminin-521 and Essential 8 medium. (A) Morphologies of forming colonies derived from 409B2 cells spiked into hMSCs are shown (images in the left). 409B2 cells (1%, 1000 cells; 0.1%, 100 cells; 0.01%, 10 cells; 0%, 0 cells) were spiked into hMSCs (100,000 cells) and co-cultured on laminin-521-coated wells in 6-well plates in Essential 8 medium for 7 days. Expression of the undifferentiated marker, TRA-1-60, in these colonies was assessed using immunofluorescence staining (images in the right). Each experiment was carried out in duplicate. Scale bars, 500 µm. (B) Numbers of the colonies detected in each spiked sample in (A) are shown. Data are present as raw data in each well (shown by plots) or the mean of well 1 and well 2 (shown by bar graphs). (C) Morphology of a forming colony derived from 253G1 cells spiked into hMSCs at the ratio of 0.001% (6 hiPSCs to 600,000 hMSCs) is shown (images in the left). Mixture of those cells was co-cultured on a 100-mm cell culture dish coated with laminin-521 in Essential 8 medium for 9 days. Forming colony was stained with anti TRA-1-60 antibody (images in the right). Experiment was carried out in duplicate. Scale bars, 500 µm.

Residual undifferentiated cells contaminating hPSC-based CTPs are a quality concern associated with tumorigencity [1]–[4]. To determine whether a culture system using laminin-521 and Essential 8 medium can detect a trace amount of undifferentiated hiPSCs in CTPs, we spiked dissociated hiPSCs into primary human somatic cells and cultured these cells on laminin-521 in Essential 8 medium. As a model of the somatic cells, we employed human mesenchymal stem cells (hMSCs), because “off-the-shelf” hMSCs derived from hPSCs are a promising CTP [12]–[14]. We spiked 409B2 cells (1%, 1000 cells; 0.1%, 100 cells; 0.01%, 10 cells) into 1×10^5^ hMSCs and plated these cells onto laminin-521-coated wells. hiPSCs were co-cultured with hMSCs on laminin-521-coated dishes in Essential 8 medium and formed distinctive colonies (Figure 4A). At day 7 after plating, we detected 362, 42.5 and 6 colonies (the mean of duplicate measurements) in 1%, 0.1% and 0.01% spiked samples, respectively (Figure 4B). We did not find any colonies when only hMSCs were cultured on laminin-521 in Essential 8 medium. In addition, immunofluorescence staining with anti-TRA-1-60 antibody showed that these colonies formed in an undifferentiated state (Figure 4A), suggesting that colonies derived from hiPSCs were formed in an hMSC monolayer under conditions with laminin-521 and Essential 8 medium. We also tested another hiPSC line, 253G1, for undifferentiated cells spiked into hMSCs. We found that 253G1 cells spiked into hMSCs at the ratio of 1% and 0.1% formed approximately 100 and 20 colonies, respectively, on laminin-521 in Essential 8 medium (Figure S4). We detected one colony when 253G1 cells were spiked into hMSCs at a ratio of 0.01% or 0.001% and co-cultured on a laminin-521-coated dish in Essential 8 medium (Figure S4 and Figure 4C). Taken together, our culture system using laminin-521 and Essential 8 medium allows the direct detection of 0.001%–0.01% hiPSCs in hMSCs as a result of efficient cell amplification. We also confirmed that no colonies were detected when a mixture of hiPSCs and hMSCs were cultured on laminin-521 in MSCGM medium instead of Essential 8 medium. In the absence of laminin-521, several colonies were detected in Essential 8 when hMSCs contained 1% hiPSCs but not when hMSCs contained 0.1% and 0.01% hiPSCs (data not shown). These results suggest that laminin-521 is required to detect trace amounts of hiPSCs in hMSCs (less than 0.1%).

**Figure 5:**
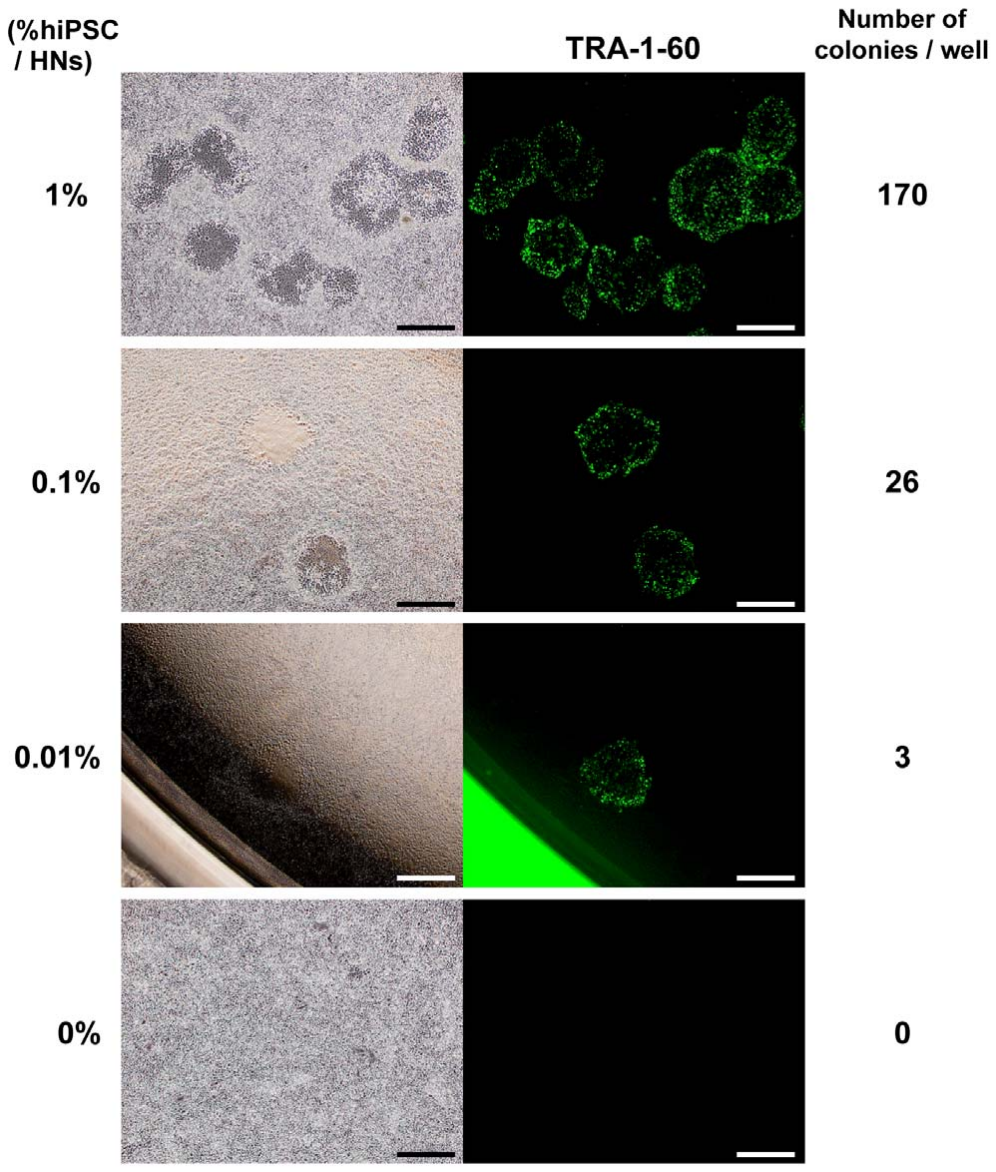
Detection of hiPSCs spiked into human neurons on the culture system using laminin-521 and Essential 8 medium. Morphologies of forming colonies derived from 253G1 cells spiked into human neurons are shown (images in the left). 253G1 cells (1%, 1000 cells; 0.1%, 100 cells; 0.01%, 10 cells; 0%, 0 cells) were spiked into human neurons (100,000 cells) and co-cultured on laminin-521-coated wells in 12-well plates in Essential 8 medium for 6 days. Forming colonies were stained with anti TRA-1-60 antibody (images in the right). HNs, human neurons. Scale bars, 500 µm.

To know whether this culture system also works in detecting trace amounts of hiPSCs contaminating other types of cells besides hMSCs, we next tested colony formation of hiPSCs spiked into primary human neurons. Spiked 253G1 cells were co-cultured with human neurons on laminin-521 in Essential 8 medium and clearly formed colonies (Figure 5), which is consistent with the observation using hiPSCs spiked into hMSCs. We detected 170, 26 and 3 colonies that were positive for TRA-1-60 when 253G1 cells were spiked into 1×10^5^ human neurons at the ratio of 1, 0.1 and 0.01%, respectively. There was no colony when only human neurons were cultured on our system. These results suggest that this culture system is also useful for detection of trace amounts of hiPSCs not only in hMSCs but also in other types of cells such as human neurons. We also confirmed that no colonies were formed on the well that was not coated with laminin-521 even when human neurons containing 10% hiPSCs were plated (data not shown), indicating that formation of the colonies derived from hiPSCs in human neurons is dependent on laminin-521.

### Culture system using laminin-521 and Essential 8 medium has a capacity for direct detection of residual undifferentiated cells contained in differentiating hiPSC cultures

**Figure 6:**
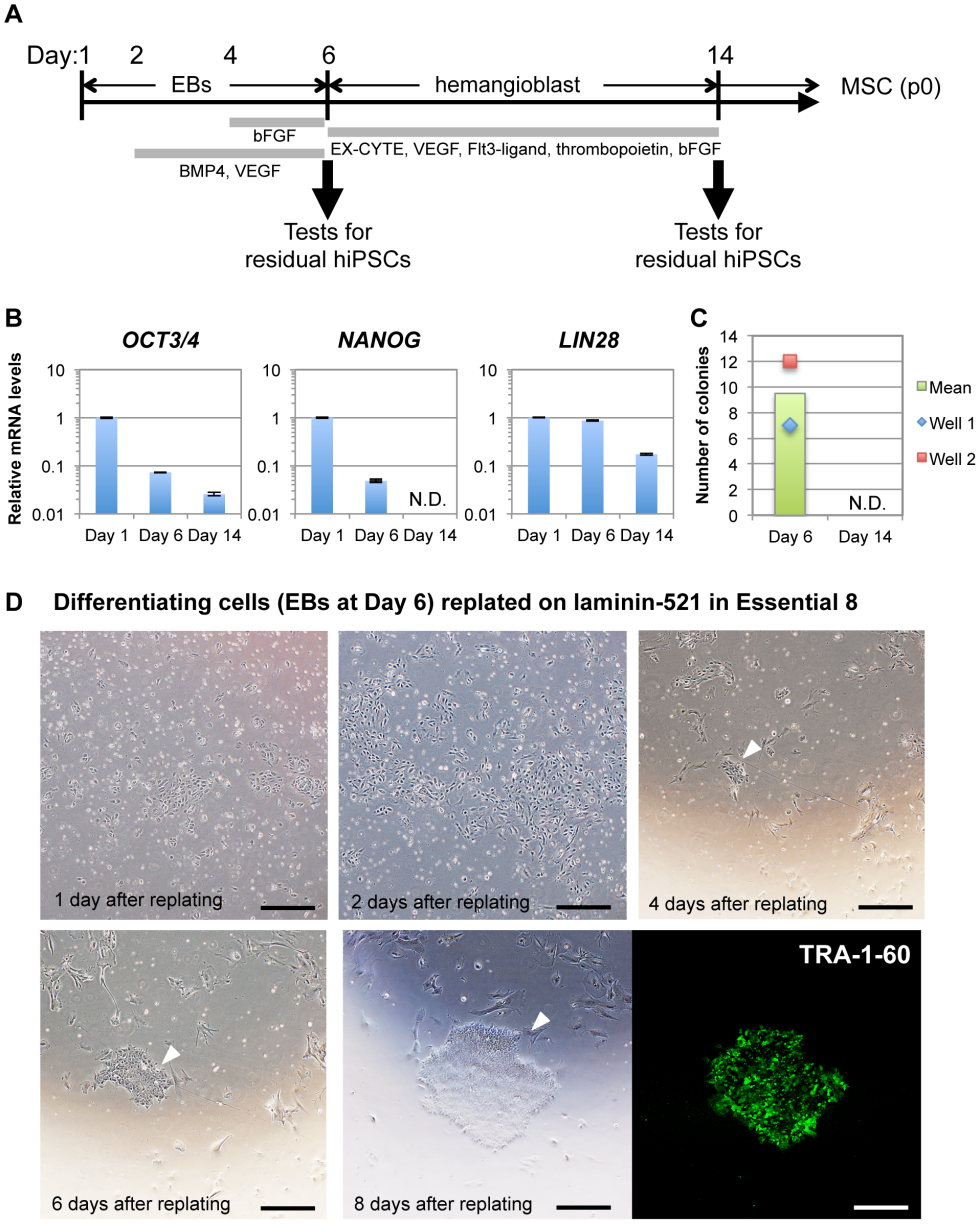
Detection of residual undifferentiated cells contained in differentiating cell cultures. (A) Differentiation scheme of 253G1 cells into MSCs is shown. (B) Expression levels of undifferentiated cell markers (*OCT3/4, NANOG* and *LIN28*) in each cell culture were determined using qRT-PCR. Relative mRNA expression levels are presented as ratios to the level of that in 253G1 cells at Day 1. Results are the mean ± SD (n = 3). (C) Numbers of the forming colonies derived from residual undifferentiated cells in differentiating cell culture at Day 6 or Day 14 are shown. Experiments were carried out in duplicate. Data are present as raw data in each well (shown by plots) or the mean of well 1 and well 2 (shown by bar graphs). (D) Phase contrast images of forming colonies derived from residual undifferentiated cells are shown. Cells at Day 6 of differentiation (EBs) were dissociated into single cells by Accutase and cultured on laminin-521-coated wells in Essential 8 medium (5×10^4^/well). After 4 days of culture, small clusters emerged and then started to grow rapidly. Finally, they formed colonies that were positive for TRA-1-60 (shown by immunofluorescence staining, green). Arrowheads indicate a colony derived from same origin. Scale bars, 500 µm.

Finally, we examined whether this culture system using laminin-521 and Essential 8 medium is applicable in direct detection of residual hiPSCs contained in differentiated cells derived from hiPSCs. We attempted to differentiate 253G1 cells into MSCs as described in Materials and Methods (Figure 6A). Using this protocol, we observed attached cells with fibroblast-like morphology at the stage of passage 0 MSCs. We confirmed that approximately 20% of these attached cells were positive for staining with anti-CD105 antibody, a MSC marker antibody (Figure S5). During the differentiation process of 253G1 cells into MSCs, we examined the expression levels of residual pluripotency markers in the cell cultures. qRT-PCR analysis revealed that expression of *OCT3/4*, *NANOG* and *LIN28* mRNA were clearly decreased in a time-dependent manner, however, expression levels at the same time point varied markedly among those genes (Figure 6B). In the cells at day 6 of differentiation, mRNA levels of *OCT3/4*, *NANOG* and *LIN28* were 7.3%, 4.8% and 86.4% of the control at day 1, respectively (Figure 6B). At day 14 of differentiation, although *OCT3/4* and *LIN28* were still at detectable levels of 2.6% and 17.2% of control cells, respectively, *NANOG* expression was not detected. These results indicate that the population of residual hiPSCs in differentiating cells, when estimated by the qRT-PCR data, greatly varies and depends on the pluripotency marker gene employed for the estimation. In addition, it is also possible that all the qRT-PCR signals were derived from partially differentiated cells, not from fully undifferentiated cells. To examine colony formation of residual undifferentiated cells in differentiating cell culture, cells at day 6 were dissociated into single cells and replated on laminin-521 in Essential 8 medium. Small cell clusters began to emerge 4 days after plating, rapidly expanded and formed colonies on laminin-521 in Essential 8, while other types of cells gradually decreased their numbers (Figure 6D). After 8 days of culture, 9.5 colonies (the mean of duplicate measurements) were formed from differentiating cells (5×10^4^) (Figure 6C) and they were all positive for TRA-1-60 (Figure 6D), indicating that the colonies were derived from residual undifferentiated cells in the differentiating cell cultures. These results suggest that the culture method using a combination of laminin-521 and Essential 8 directly detects residual undifferentiated cells by highly efficient cell amplification. Based on our finding that approximately 0.3 and 6.7 colonies were formed from 1×10^4^ MSCs containing 0.01% and 0.1% of 253G1 cells, respectively, in this culture system (Figure S4), and assuming that the sensitivity of the system for hPSCs in EBs are comparable to that in MSCs, the population of the undifferentiated cells in the differentiating cell cultures on day 6 (1.9 colonies/10^4^ cells) was estimated to be in between 0.01% and 0.1%. When we tested colony formation using cell cultures on day 14 of differentiation, no colonies were detected on laminin-521 in Essential 8 medium (Figure 6C and data not shown), suggesting that the population of the residual hiPSCs was less than 0.01%.

## Discussion

A method to detect residual undifferentiated hPSCs contained in CTPs is required to evaluate product quality during manufacturing processes. In the present study, we propose a novel method to detect a trace amount of undifferentiated hPSCs by highly efficient amplification of those cells *in vitro*. We showed that Essential 8 medium significantly promotes cell growth of hiPSCs dissociated into single cells on laminin-521 compared with the conventional medium, mTeSR1. In addition, Essential 8 medium allowed robust proliferation of hiPSCs even at low cell density on laminin-521. We also demonstrated that 0.001%–0.01% hiPSCs spiked into primary hMSCs were clearly detected and formed colonies on laminin-521 in Essential 8. Similarly, we confirmed that 0.01% hiPSCs spiked into primary human neurons were also detectable on this system. Moreover, we showed that residual undifferentiated hiPSCs contained in differentiating cells were detectable by forming colonies on laminin-521 in Essential 8 in the process of hMSC differentiation. These results indicate that a culture system utilizing a combination of laminin-521 and Essential 8 medium provides a direct and highly sensitive method for detecting undifferentiated hPSCs. To our knowledge, this is the first report to show a direct and highly-sensitive *in vitro* method for detecting undifferentiated hPSCs as impurities in CTPs.

In this study, highly efficient amplification of undifferentiated hPSCs has been uniquely applied to quality control of CTPs. Amplified hPSC colonies were visible using phase-contrast microscopy and also immunofluorescence staining using pluripotency antibodies, which enabled direct detection of hPSCs contaminating CTPs. Our method distinguished between undifferentiated cells and other cells *in vitro*, and overcame the disadvantage of other *in vitro* methods such as flow cytometry and qRT-PCR. The flow cytometry analysis detects known marker molecules expressed in undifferentiated hPSCs using antibodies and proteins. Signals originating from non-specific detection commonly affect sensitivity of the assay as background. Our *in vitro* method can lower the background arising from non-specific detection and is expected to specifically detect residual undifferentiated hPSCs in CTPs. The qRT-PCR method is highly sensitive and can rapidly quantify undifferentiated cell contamination in CTPs. However, in the present study, gene expression levels of pluripotency markers during the differentiation process of hiPSCs into MSCs varied markedly among those marker genes (Figure 6B). Moreover, there remains a possibility that expression signals of marker genes were not derived from totally undifferentiated hPSCs, but from partially differentiated cells. Indeed, the expression level of *LIN28* did not decrease so much during the differentiation as those of the other genes, which was not obviously associated with the differentiation status of the cells in EBs on Day 6 (Figure 6B), although we have previously reported that *LIN28* was a useful marker for monitoring the level of residual hiPSCs in RPE cells derived from hiPSCs [3]. Thus, it is difficult to determine the presence of residual hiPSCs simply by qRT-PCRs. In contrast, direct detection method using the highly efficient amplification system can clearly detect the presence of intact undifferentiated cells. Based on the result from direct detection of residual hiPSCs when tested the cells on Day 6 of differentiation (approximately 0.01%–0.1%) (Figure 6C-D), it is conceivable that the qRT-PCR signals for the pluripotency marker genes (Figure 6B) are partly derived from residual hiPSCs but mainly derived from partially differentiated cells. Similarly, in the case of the cells at Day 14 of differentiation, the majority of the qRT-PCR signals of *OCT3/4* and *LIN28* (Figure 6B) are considered to be attributable to partially differentiating cells but not to intact hiPSCs. Combination of the *in vitro* methods including our cell culture method would mutually support useful quality assessment of CTPs to detect undifferentiated hPSCs.

In addition to the detection of undifferentiated cells, this culture system using laminin-521 and Essential 8 medium allows further characterization of the undifferentiated cells if they are maintained *in vitro* or inoculated into immunodeficient animals. Analyses for the properties of the residual undifferentiated cells would be necessary not only for the quality assessment of CTPs, but also for improvement of quality specifications of hPSCs as a raw/intermediate material for production of CTPs.

Here, we showed that our culture system is able to detect 0.01% of 409B2 hiPSCs and 0.001% of 253G1 hiPSCs, both of which were spiked into hMSCs (Figure 4). The detection sensitivity for hiPSCs spiked into hMSCs was different between the two hiPSC lines, although such a difference in cell growth on laminin-521 was not found between these two cell lines (Figure 3). This difference may be attributable to the difference in the growth potential of hPSCs in the specific environment provided by CTPs. Kanemura *et al.* have recently demonstrated that hiPSCs co-cultured with iPSC-derived RPE undergo apoptosis by pigment epithelium-derived factor (PEDF) secreted from hiPSC-derived RPE [15], showing that CTPs themselves have the potential to affect cell growth of hPSCs. In the present study, the influence of the co-culture system with hMSCs to the proliferation of hiPSCs might have been different between the two cell lines.

The mechanism by which laminin-521 and Essential 8 medium enhance hiPSCs cell proliferation remains unclear. Rodin *et al.* have recently shown that addition of E-cadherin to laminin-521 permitted the efficient clonal expansion of hESCs [7]. E-cadherin is known to be the primary cell-cell adhesion molecule and essential for hESC survival [16]. We observed that anti-E-cadherin antibody decreased growth potential of hiPSCs under our experimental conditions (data not shown). Therefore, E-cadherin signaling may play some important roles in the rapid cell growth on laminin-521 in Essential 8 medium.

Tumorigenicity is one of the major safety concerns for CTPs derived from hPSCs that are transplanted into patients. However, testing strategies for the tumorigenicity of hPSC-derived CTPs have not yet been established. Here, we introduced a novel testing method for directly detecting a trace amount of undifferentiated hPSCs *in vitro*. The ability of each tumorigenicity-associated test should be taken into consideration to evaluate tumorigenicity of residual undifferentiated hPSCs as impurities in products. *In vivo* tumorigenicity tests using immunodeficient animals can detect tumorigenic cells including undifferentiated hPSCs, but this method is costly and time-consuming. The flow cytometry analysis and qRT-PCR are rapid, but these methods indirectly detect tumorigenic cells depending on marker molecules. Risk of tumorigenicity in hPSCs-derived CTPs should be assessed, based on the results from an appropriate combination of these tumorigenicity-associated tests. Our novel method will contribute to establishment of the testing strategies for tumorigenicity in products, following evaluation of the quality of CTPs derived from hPSCs for the future regenerative medicine/cell therapy.

## Supporting Information

Figure S1 (A) Quantification of the number of dissociated 201B7 cells expanded on laminin-521 or Matrigel in Essential 8 or mTeSR1 medium. Data are presented as the mean ± standard deviation (SD) of three independent experiments (**P<0.01, two-way ANOVA followed by a Bonferroni post-hoc test). LN521, laminin-521. MG, Matrigel. (B) Quantification of the number of dissociated 201B7 cells expanded on laminin-521 or LM511-E8 in Essential 8 or mTeSR1 medium. Results are presented as the mean ±SD (n = 3) (***P<0.001, two-way ANOVA followed by a Bonferroni post-hoc test).
(TIF)

Figure S2 (A-B) Expression levels of undifferentiated markers (*OCT3/4, NANOG, SOX2* and *LIN28*) in 201B7 cells (A) and 409B2 cells (B) subcultured on laminin-521 in Essential 8 were determined using qRT-PCR. Relative mRNA expression levels are presented as ratios to the level of that in control cells subcultured on Matrigel in mTeSR1 medium by colony passage. Results are presented as the mean ± SD (n = 3). (C-D) Expression levels of markers for the differentiation of embryoid bodies (EBs) derived from 201B7 cells (C) and 409B2 cells (D): endoderm (*GATA6, SOX17*), mesoderm (*CDH5, FOXF1*), and ectoderm (*SOX1, PAX6*). Relative mRNA expression levels are presented as ratios to the level of that in control cells (EBs at day 10). Results are presented as the mean ± SD (n = 3).
(TIF)

Figure S3 **Quantification of the number of 253G1 cells expanded on laminin-521 in Essential 8 or mTeSR1 medium.** Cell numbers were counted at day 6, 9, and 12 after plating at 8.0×10^3^ cells/cm^2^ or 8.0×10^2^ cells/cm^2^.
(TIF)

Figure S4 **Morphologies of forming colonies derived from 253G1 cells spiked into hMSCs are shown (images in the left).** 253G1 cells (1%, 300 cells; 0.1%, 30 cells; 0.01%, 3 cells; 0%, 0 cells) were spiked into hMSCs (30,000 cells) and co-cultured on 12-well plates coated with laminin-521 in Essential 8 medium for 9 days. Expression of the undifferentiated cell marker, TRA-1-60, in these colonies was assessed using immunofluorescence staining (images to the right). Each experiment was carried out in duplicate.
(TIF)

Figure S5 **Phase contrast images of the cells at day 18 of differentiation (at the stage of passage 0 MSCs) are shown.** Expression of MSC marker, CD105, in these cells was examined using immunofluorescence staining (images to the right). Arrowheads indicate the cells that were positive for CD105.
(TIF)

Table S1 **Sequences of the primers and probes for qRT-PCR.**
(DOCX)
