# Supplementary figures and images for: A Novel In Vitro Method for Detecting Undifferentiated Human Pluripotent Stem Cells as Impurities in Cell Therapy Products Using a Highly Efficient Culture System
Source: PLoS One. 2014 Oct 27;9(10):e110496. doi: 10.1371/journal.pone.0110496 (PMC4210199; doi:10.1371/journal.pone.0110496)

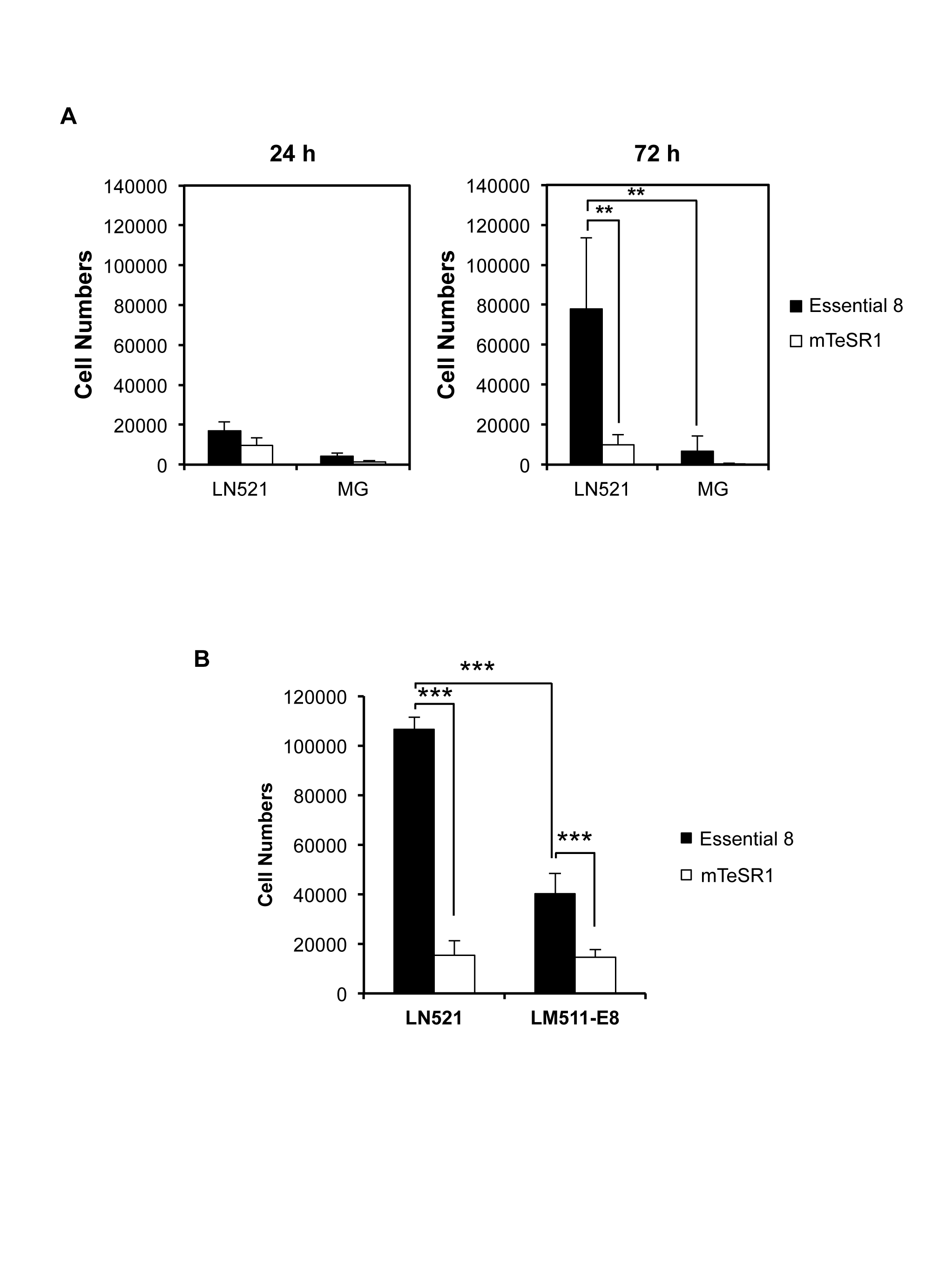

Supplement: Figure S1 — (A) Quantification of the number of dissociated 201B7 cells expanded on laminin-521 or Matrigel in Essential 8 or mTeSR1 medium. Data are presented as the mean ± standard deviation (SD) of three independent experiments (**P<0.01, two-way ANOVA followed by a Bonferroni post-hoc test). LN521, laminin-521. MG, Matrigel. (B) Quantification of the number of dissociated 201B7 cells expanded on laminin-521 or LM511-E8 in Essential 8 or mTeSR1 medium. Results are presented as the mean ±SD (n = 3) (***P<0.001, two-way ANOVA followed by a Bonferroni post-hoc test). (TIF) [file pone.0110496.s001.tif]

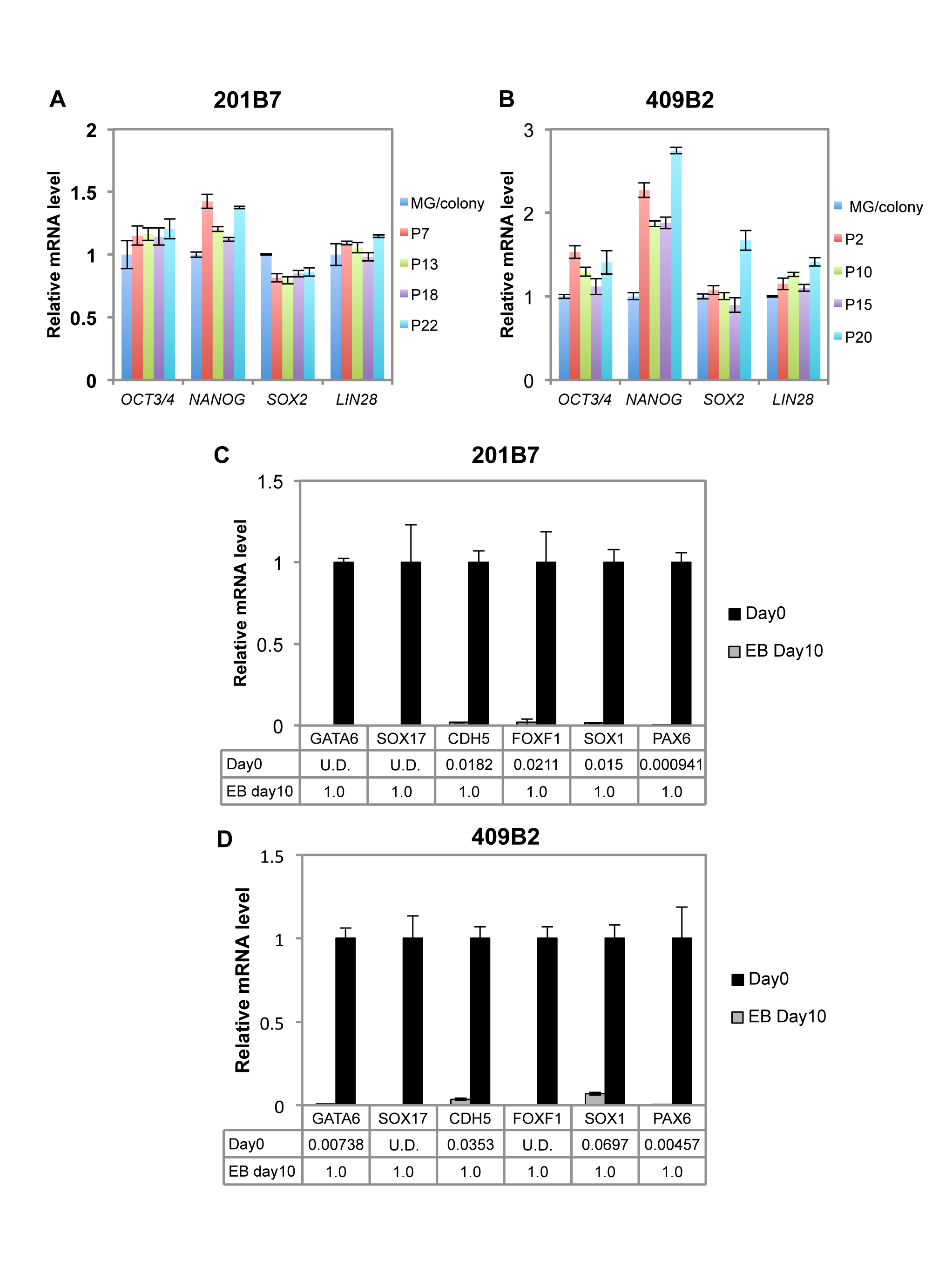

Supplement: Figure S2 — (A-B) Expression levels of undifferentiated markers (OCT3/4, NANOG, SOX2 and LIN28) in 201B7 cells (A) and 409B2 cells (B) subcultured on laminin-521 in Essential 8 were determined using qRT-PCR. Relative mRNA expression levels are presented as ratios to the level of that in control cells subcultured on Matrigel in mTeSR1 medium by colony passage. Results are presented as the mean ± SD (n = 3). (C-D) Expression levels of markers for the differentiation of embryoid bodies (EBs) derived from 201B7 cells (C) and 409B2 cells (D): endoderm (GATA6, SOX17), mesoderm (CDH5, FOXF1), and ectoderm (SOX1, PAX6). Relative mRNA expression levels are presented as ratios to the level of that in control cells (EBs at day 10). Results are presented as the mean ± SD (n = 3). (TIF) [file pone.0110496.s002.tif]

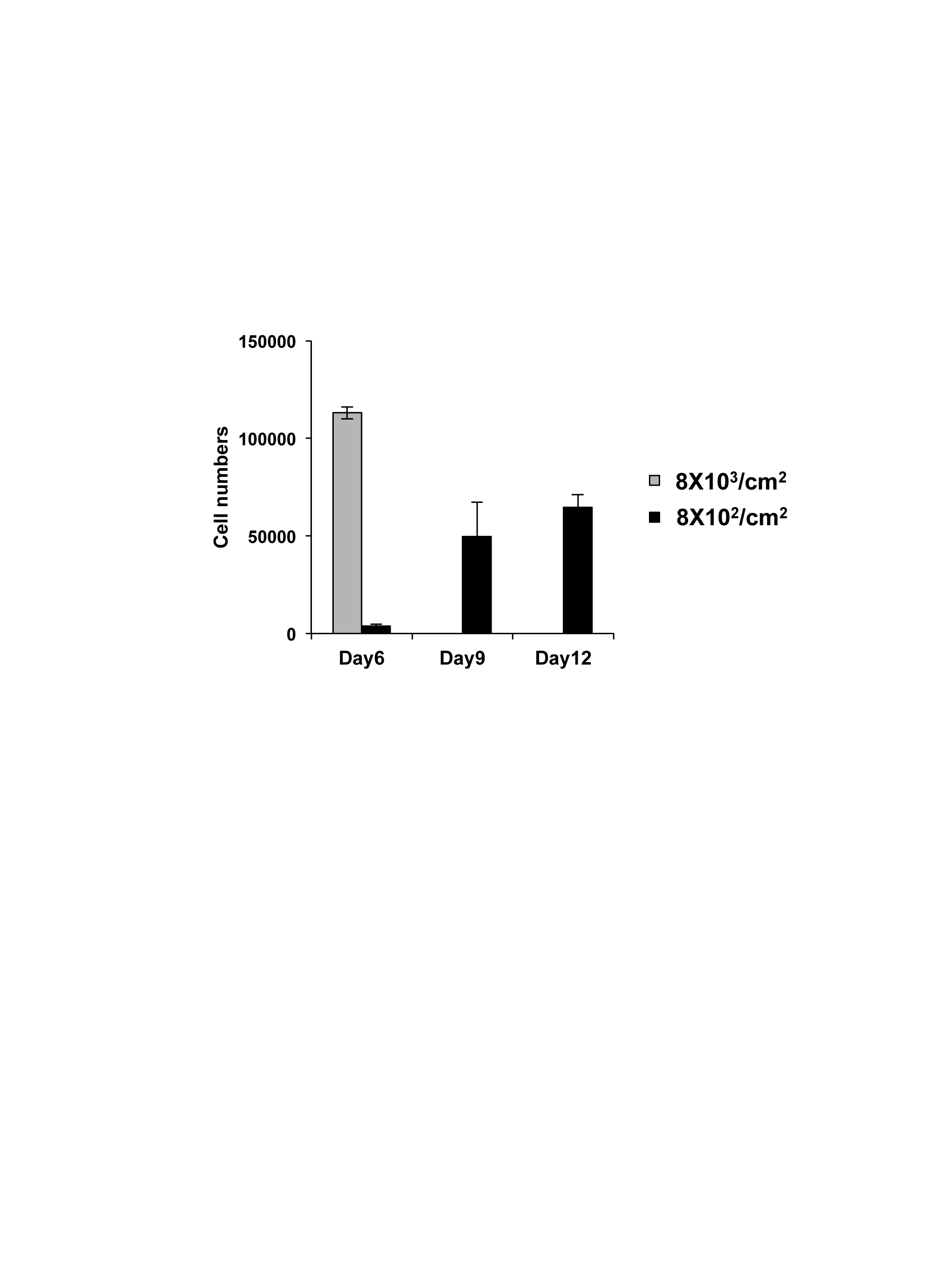

Supplement: Figure S3 — Quantification of the number of 253G1 cells expanded on laminin-521 in Essential 8 or mTeSR1 medium. Cell numbers were counted at day 6, 9, and 12 after plating at 8.0×10^3^ cells/cm2 or 8.0×10^2^ cells/cm2. (TIF) [file pone.0110496.s003.tif]

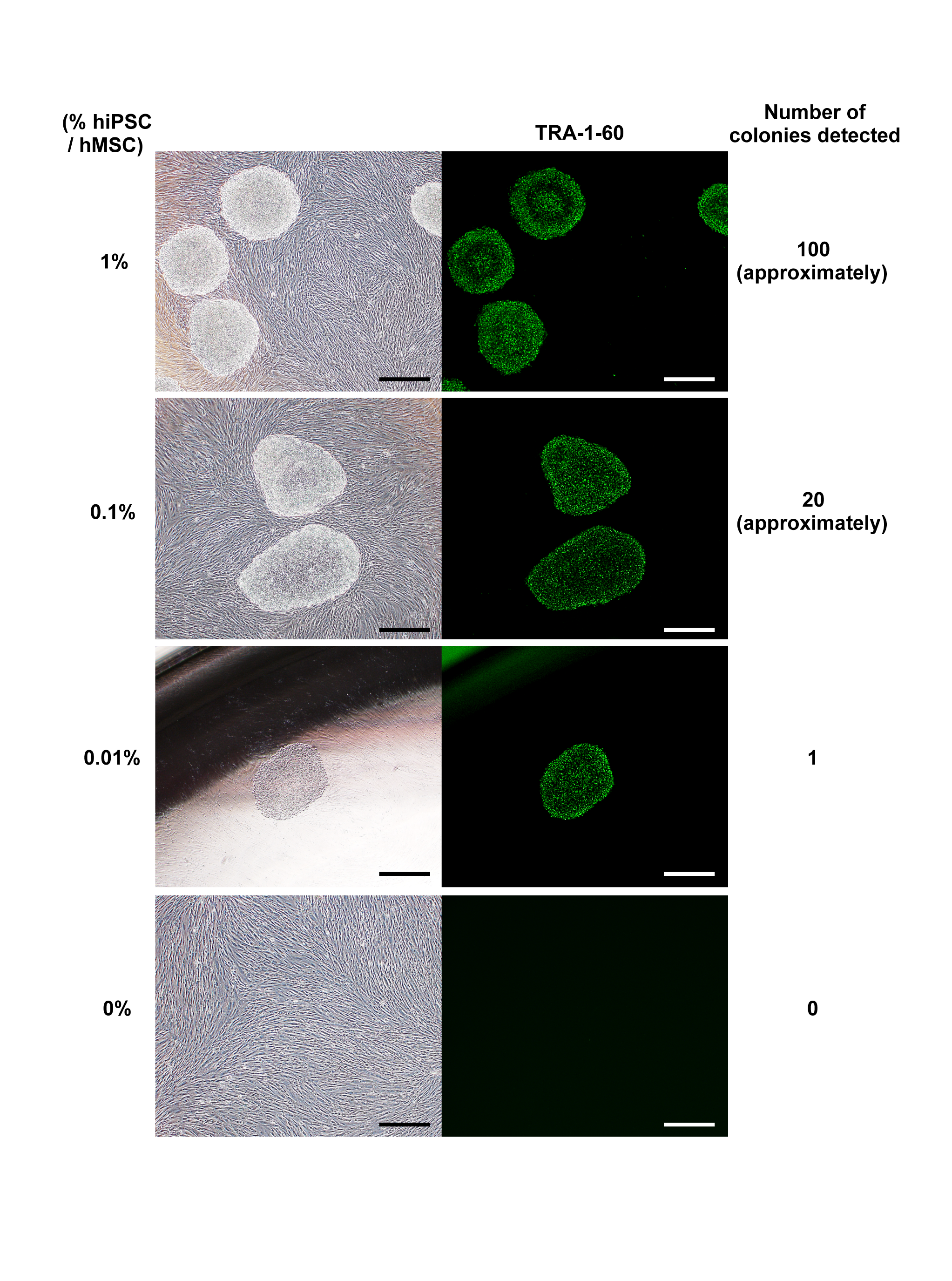

Supplement: Figure S4 — Morphologies of forming colonies derived from 253G1 cells spiked into hMSCs are shown (images in the left). 253G1 cells (1%, 300 cells; 0.1%, 30 cells; 0.01%, 3 cells; 0%, 0 cells) were spiked into hMSCs (30,000 cells) and co-cultured on 12-well plates coated with laminin-521 in Essential 8 medium for 9 days. Expression of the undifferentiated cell marker, TRA-1-60, in these colonies was assessed using immunofluorescence staining (images to the right). Each experiment was carried out in duplicate. (TIF) [file pone.0110496.s004.tif]

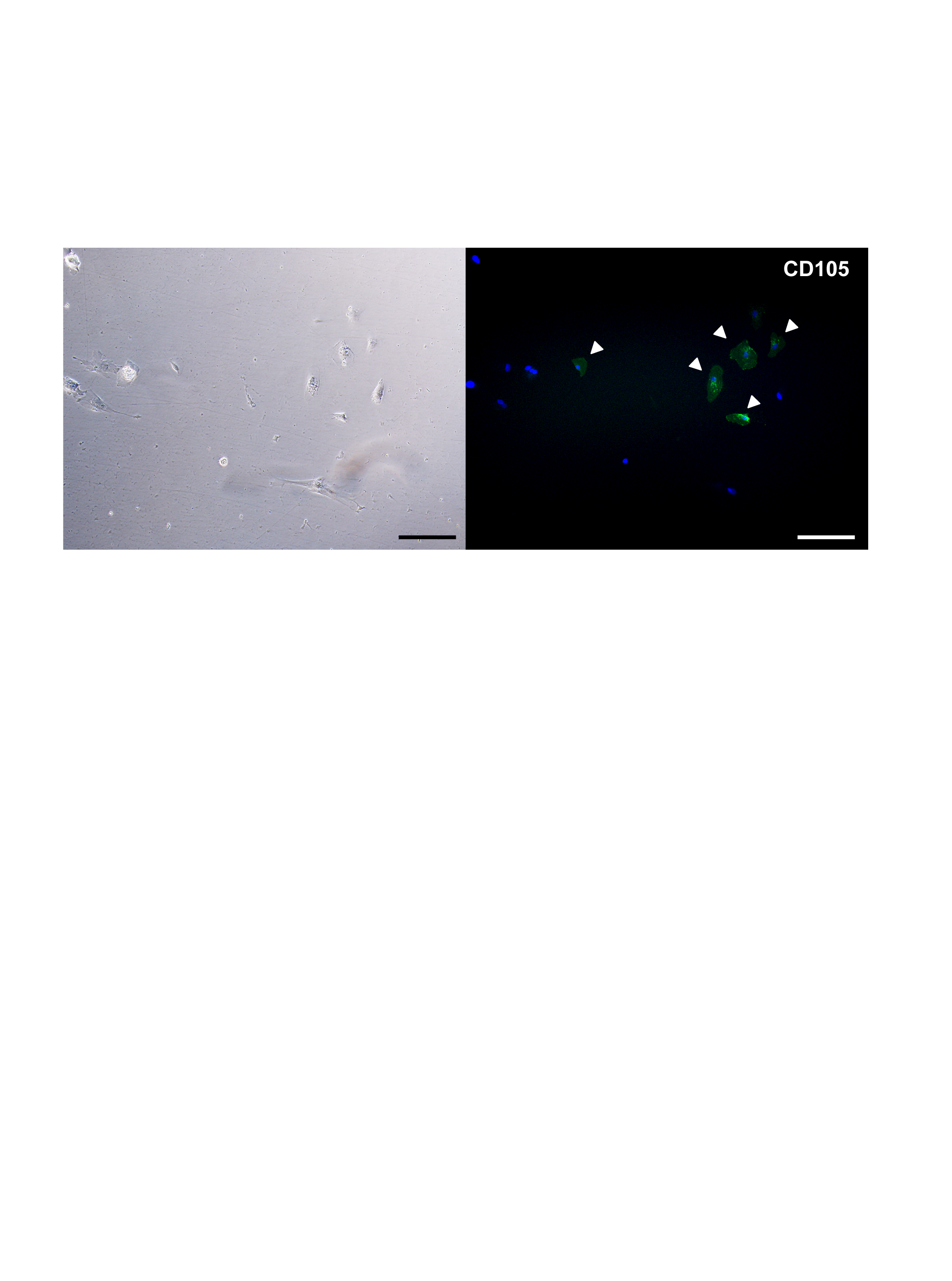

Supplement: Figure S5 — Phase contrast images of the cells at day 18 of differentiation (at the stage of passage 0 MSCs) are shown. Expression of MSC marker, CD105, in these cells was examined using immunofluorescence staining (images to the right). Arrowheads indicate the cells that were positive for CD105. (TIF) [file pone.0110496.s005.tif]
